# Supplementary material for: Identification of photoperiod-induced specific miRNAs in the adrenal glands of Sunite sheep (Ovis aries)
Source: Front Vet Sci. 2022 Jul 22;9:888207. doi: 10.3389/fvets.2022.888207 (PMC9354845; doi:10.3389/fvets.2022.888207)
Supplement: Supplementary file 1 [file Data_Sheet_1.ZIP › Supplementary file/miRDeep2 file.zip!/miRDeep2 file/Novel_Structure/SPLP42A3/Novel_580.pdf]

Provisional ID	:	NC_019458.2_2952
Score total	:	4.8
Score for star read(s)	:	3.9
Score for read counts	:	-2.8
Score for mfe	:	2.2
Score for randfold	:	1.6
Score for cons. seed	:	
Total read count	:	6
Mature read count	:	4
Loop read count	:	0
Star read count	:	2

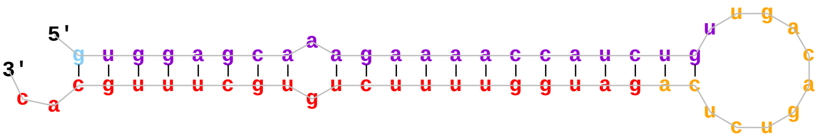

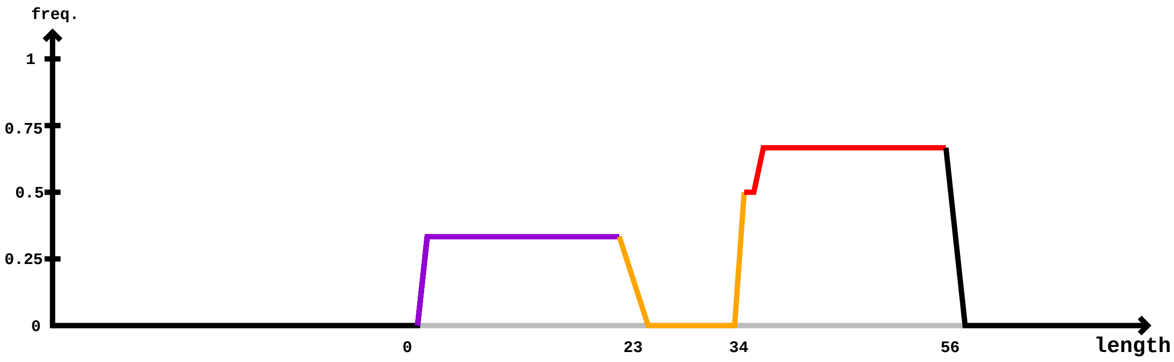

Star **Mature**

```
5'- aucuaggcaguuuggaaagaggugggga gugugugug
```

```
5'- aucuaggcaguuuggaaagagguggggagugugugug uggagcaaagaaaaccaucugu ugacagucuca gaugguuuucugugcuuugcac guccucucuccccagcacug  -3'   obs
    aucuaggcaguuuggaaagagguggggagugugugu guggagcaaagaaaaccaucug uugacagucuca gaugguuuucugugcuuugcac guccucucuccccagcacug        exp
    .(((((.....))))).((.(.(((((((.(.((((((((((((.(((((((((((((.........))))))))))))).)))))))))))).)..).))))))).).)).   reads   mm   sample
    .....................................uggagcaaagaaaaccaucugu.....................................................   2       0    seq
    ......................................................................gaugguuuucugugcuuugcac....................   3       0    seq
    ........................................................................ugguuuucugugcuuugcacg...................   1       0    seq
```
